# The novel BH3 α-helix mimetic JY-1-106 induces apoptosis in a subset of cancer cells (lung cancer, colon cancer and mesothelioma) by disrupting Bcl-xL and Mcl-1 protein–protein interactions with Bak

**DOI:** 10.1186/1476-4598-12-42

**Published:** 2013-05-16

**Authors:** Xiaobo Cao, Jeremy L Yap, M Karen Newell-Rogers, Chander Peddaboina, Weihua Jiang, Harry T Papaconstantinou, Dan Jupitor, Arun Rai, Kwan-Young Jung, Richard P Tubin, Wenbo Yu, Kenno Vanommeslaeghe, Paul T Wilder, Alexander D MacKerell, Steven Fletcher, Roy W Smythe

**Affiliations:** 1 Department of Surgery, Scott & White Memorial Hospital and Clinic, The Texas A&M University System, Health Science Center, College of Medicine, 702 SW HK Dodgen Loop, Temple, Texas 76504, USA; 2 Department of Pharmaceutical Science, University of Maryland School of Pharmacy, 20 N. Pine St., Baltimore, MD 21201, USA; 3 Department of Biochemistry and Molecular Biology, University of Maryland School of Medicine, 108 N. Greene St., Baltimore, MD 21201, USA; 4 University of Maryland Marlene and Stewart Greenebaum Cancer Center, 22 S. Greene St., Baltimore, MD 21201, USA

**Keywords:** Mcl-1, Bcl-xL, Small molecule inhibitor, Cancer, BH3 mimetic

## Abstract

**Background:**

It has been shown in many solid tumors that the overexpression of the pro-survival Bcl-2 family members Bcl-2/Bcl-xL and Mcl-1 confers resistance to a variety of chemotherapeutic agents. We designed the BH3 α-helix mimetic JY-1-106 to engage the hydrophobic BH3-binding grooves on the surfaces of both Bcl-xL and Mcl-1.

**Methods:**

JY-1-106–protein complexes were studied using molecular dynamics (MD) simulations and the SILCS methodology. We have evaluated the in vitro effects of JY-1-106 by using a fluorescence polarization (FP) assay, an XTT assay, apoptosis assays, and immunoprecipitation and western-blot assays. A preclinical human cancer xenograft model was used to test the efficacy of JY-1-106 in vivo.

**Results:**

MD and SILCS simulations of the JY-1-106–protein complexes indicated the importance of the aliphatic side chains of JY-1-106 to binding and successfully predicted the improved affinity of the ligand for Bcl-xL over Mcl-1. Ligand binding affinities were measured via an FP assay using a fluorescently labeled Bak-BH3 peptide *in vitro*. Apoptosis induction via JY-1-106 was evidenced by TUNEL assay and PARP cleavage as well as by Bax–Bax dimerization. Release of multi-domain Bak from its inhibitory binding to Bcl-2/Bcl-xL and Mcl-1 using JY-1-106 was detected via immunoprecipitation (IP) western blotting.

At the cellular level, we compared the growth proliferation IC_50_s of JY-1-106 and ABT-737 in multiple cancer cell lines with various Bcl-xL and Mcl-1 expression levels. JY-1-106 effectively induced cell death regardless of the Mcl-1 expression level in ABT-737 resistant solid tumor cells, whilst toxicity toward normal human endothelial cells was limited. Furthermore, synergistic effects were observed in A549 cells using a combination of JY-1-106 and multiple chemotherapeutic agents. We also observed that JY-1-106 was a very effective agent in inducing apoptosis in metabolically stressed tumors. Finally, JY-1-106 was evaluated in a tumor-bearing nude mouse model, and was found to effectively repress tumor growth. Strong TUNEL signals in the tumor cells demonstrated the effectiveness of JY-1-106 in this animal model. No significant side effects were observed in mouse organs after multiple injections.

**Conclusions:**

Taken together, these observations demonstrate that JY-1-106 is an effective pan-Bcl-2 inhibitor with very promising clinical potential.

## Background

Despite decades of cancer research, the survival rates for patients with solid tumors have improved only modestly. Many tumors are unresponsive to conventional therapy due to the resistance of tumor cells to apoptosis, or programmed cell death. Since the molecular cloning of Bcl-2 [1], the anti-apoptotic members of the Bcl-2 family, which include Bcl-2, Bcl-xL and Mcl-1, have been identified as key regulators of mitochondria membrane potential and oncogenesis, as well as chemoresistance [2].

Bcl-xL was found to have a unique role in chemoresistance in multiple cancers in an NIH Developmental Therapeutics Program study that determined that high levels of Bcl-xL protect a variety of cancer cell lines from 70,000 cytotoxic agents [2]. The downregulation of Bcl-xL has been shown to induce apoptosis and increase chemosensitivity. ABT-737 [3], the most well-known member of a class of Bcl-2-family targeting compounds, and its orally active analog ABT-263 [4], have activity as single agents in a subset of cancers (including multiple myeloma and small-cell lung cancer) that rely on Bcl-2/Bcl-xL, but not Mcl-1, for survival.

Because of the overexpression and overlapping functions of the Bcl-2 family proteins, Mcl-1 can compensate for the loss of the anti-apoptotic function of Bcl-2/xL. Recent studies demonstrated that cancer cells rapidly develop resistance to ABT-737 through the up-regulation of Mcl-1 and that the down-regulation of Mcl-1 restores the sensitivity to ABT-737 [5,6]. Mcl-1 reduction significantly enhances the sensitivity of cancer cells to ABT-737 and other chemotherapeutics [6,7]. Hence, these findings suggest that Mcl-1 overexpression may function as an additional survival mechanism to protect cancer cells against conventional therapies.

Although the basic topology of BH3 domain hydrophobic binding groove is highly conserved among the prosurvival Bcl-2 family members such as Bcl-2, Bcl-xL and Mcl-1 [8], there is a selectivity in binding defined by the specific pattern of amino acid side chains located on the α2, α4, and α5 helices [7]. This may explain why ABT-737 does not exhibit potency against Mcl-1. Because this hydrophobic groove normally accommodates the BH3 domain of pro-apoptotic Bcl-2 proteins, it has been hypothesized that small molecules that bind to this BH3-binding groove in Bcl-2, Bcl-xL, or Mcl-1 may be capable of blocking their heterodimerization with a subset of pro-apoptotic members in the Bcl-2 protein family, such as Bax, Bid, and Bak. This would expand the pool of free pro-apoptotic effectors and, thus, induce apoptosis in cancer cells in which overexpressed Bcl-2, Bcl-xL, or Mcl-1 provide survival cues. Hence, the development of BH3 mimetics could be a feasible and clinically effective approach to simultaneously inhibiting Bcl-2/xL and Mcl-1 functions.

Indeed, several non-peptidic small-molecule BH3 mimetics designed to bind key domains in the hydrophobic BH3-binding groove have already been identified [9,10], the most extensively studied of which is the previously mentioned compound ABT-737. An alternative strategy to the disruption of this protein–protein interaction centers on the observation that the BH3 domains of the pro-apoptotic proteins become α-helical upon binding their anti-apoptotic partners [11]. Accordingly, small-molecules have been designed to reproduce the relative projections of key hydrophobic side chains found on one face of the BH3 α-helix. For example, mimicry of Val74, Leu78, Ile81 (and Ile85) on one face of the Bak-BH3 α-helix has afforded potent Bcl-xL inhibitors [12]. More recently, an α-helix mimetic strategy based on a terphenyl scaffold has furnished a “pan-Bcl-2” antagonist, inhibiting Bcl-2, Bcl-xL and Mcl-1 [13]. However, many of the BH3 mimetics that potently engage the Bcl-2/Bcl-xL/Bcl-w sub-class of the anti-apoptotic Bcl-2 proteins often only weakly inhibit members of the Mcl-1/Bfl-1 sub-class. An effective BH3 mimetic should “neutralize” both sub-classes, as this is required for apoptosis to occur.

We herein describe the biological characterization of our novel “pan-Bcl-2” inhibitor JY-1-106, which, based on a trisarylamide framework, reproduces the chemical nature and relative spatial projections of the key hydrophobic side chains on one face of the BH3 α-helix. JY-1-106 induces cancer cell death regardless of the Mcl-1 expression levels through intrinsic apoptosis pathways, and sensitizes tumor cells to chemotherapeutic agents and to metabolic stress. Furthermore, we demonstrate that JY-1-106 inhibits tumor growth in a lung cancer xenograft model, and, therefore, that α-helix mimicry based on a trisarylamide scaffold warrants further investigation towards the development of novel chemotherapeutics.

## Results

### Design

**Figure 1:**
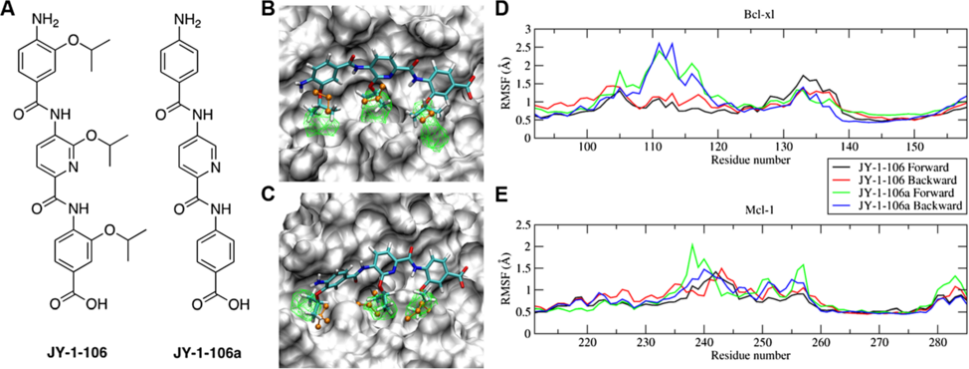
**The computational design of JY-1-106.** (**A**) Chemical structures of JY-1-106 and its analog JY-1-106a. (**B** and **C**) Probability distribution maps of JY-1-106 (forward orientation) side chain carbon atoms (green) along with the corresponding crystallographic peptide side chain heavy atoms (orange) overlaid on a representative conformation of JY-1-106 determined from the MD simulation for both Bcl-xL (**B**) and Mcl-1 (**C**). RMS fluctuations of residues lining the binding pocket for Bcl-xL (**D**) and Mcl-1 (**E**), with JY-1-106 and JY-1-106a in both the forward and backward orientations.

Both “faces” of the BH3 α-helix contribute to the stabilization of the protein–protein complex upon docking with the BH3-binding groove. In addition to the previously mentioned hydrophobic residues on one face of the Bak-BH3 α-helix, Arg76 and Asp83 located on the other face of the helix are also important for binding [11]. Thus, towards the development of potent, pan-Bcl-2 antagonists, we wished to design amphipathic α-helix mimetics that would achieve more superior α-helix mimicry than ever reported before, as well as, potentially, better selectivity profiles against non-Bcl-2 proteins. We reasoned that this process would be accelerated by selecting and modifying a functional α-helix mimetic from the literature. Compounds based on an oligoamide-foldamer strategy appeared excellent candidates, primarily owing to their straightforward chemical syntheses [14]. A structure–activity relationship analysis of the backbone of a previously reported oligoamide-based α-helix mimetic designed to inhibit Bcl-xL [14] led to the discovery of the novel compound JY-1-106 (Figure 1A) with even greater affinity for Bcl-xL [15]. Although only the second most potent compound of the congeners synthesized, the aqueous solubility of JY-1-106 was, in our hands, greater than that of the most potent derivative, and so JY-1-106 was selected for further biological characterization.

### Computational analyses of the binding of JY-1-106 to Bcl-xL and Mcl-1

Molecular details of the interactions of JY-1-106 with Bcl-xL and Mcl-1 were obtained by modeling inhibitor binding with these proteins based on the crystallographic orientations of the bound peptides, followed by MD simulations. In addition, the SILCS methodology [16,17] was applied to quantify the energetic differences associated with binding to the two proteins and between the binding of JY-1-106 and its analog JY-1-106a (Figure 1A) to the proteins. Analysis of the MD sampled complex conformations suggested that the JY-1-106 binds to Bcl-xL and Mcl-1 in the same way as Bak, Bax and other BH3 peptides. From the MD simulations, 3D probability distributions of the carbon atoms in the three aliphatic side chains of JY-1-106 (forward orientation) were obtained and are presented in Figures 1B and 1C for Bcl-xL and Mcl-1, respectively, along with the positions of the corresponding amino acid side chains from the BH3-protein crystal structures and a representative orientation of JY-1-106 from the MD simulation. The hydrophobic interactions between the BH3 peptide and the protein were reproduced by JY-1-106 quite well as indicated by the overlap between the probability distributions and the experimental BH3 peptide side chain positions.

To further examine the role of the aliphatic functional groups of JY-1-106 in protein binding, simulations of JY-1-106a (Figure 1A) were also performed to compare with simulations of JY-1-106. For Bcl-xL, much larger flexibilities occur for residues between 105 and 120 when JY-1-106a is bound versus JY-1-106 (Figure 1D), and higher flexibilities for residues between 250 and 260 also occur for Mcl-1 when JY-1-106a is present (Figure 1E). Previously, it was observed that residues between 105 and 120 of Bcl-xL have higher flexibilities in the apo form compared with the peptide bound form [18]. Additionally, residues between 250 and 260 have higher flexibilities when the bound peptide is absent for Mcl-1, consistent with previous observations [12]. The RMSF plots in our current study suggest that the protein structure is closer to the apo-form when JY-1-106a is present and closer to the peptide bound form when JY-1-106 is present for both Bcl-xL and Mcl-1. This emphasizes the role of the hydrophobic side chains in JY-1-106 for binding.

Subsequent calculations applied the SILCS methodology [16,17] to estimate binding affinities based on ligand grid free energy (LGFE) scores were calculated to quantify the binding of JY-1-106 to the two proteins using three different approaches (Table 1). The two less computationally demanding LGFE approaches give similar LGFE scores: approximately −10 kcal/mol for JY-1-106 binding to Bcl-xL and about −7 kcal/mol for Mcl-1. LGFE scores calculated using the conformations from the 50 ns MD simulations give more favorable scores of approximately −14 and −8 kcal/mol for Bclxl and Mcl-1, respectively. Thus, the SILCS methodology predicts the JY-1-106 to interact more favorably with Bcl-xL versus Mcl-1 by a range of 2 to 8 kcal/mol depending on the methodology, consistent with the experimental analysis presented below. Notably, the LGFE scores obtained for forward and backward orientations of JY-1-106 are similar, suggesting that both binding orientations are possible.

**Table 1:** Calculation of LGFEs of JY-1-106

**Total LGFEs**		**Minimized**		**Langevin dynamics**		**Explict solvent MD**	
Bcl-xL							
	F	-10.2 (4.3)		-10.6 (4.2)		-13.8 (4.1)	
	B	-10.1 (3.9)		-10.6 (4.0)		-15.8 (2.7)	
Mcl-1							
	F	-7.3 (2.9)		-7.2 (2.3)		-8.7 (1.5)	
	B	-7.8 (3.9)		-7.0 (2.2)		-7.5 (2.2)	
Difference, Mcl-1 - Bcl-xL							
	F	2.9		3.4		5.1	
	B	2.3		3.6		8.3	
Group contributions		Minimized		Langevin dynamics		Explict Solvent MD	
		Aromatic	Aliphatic	Aromatic	Aliphatic	Aromatic	Aliphatic
Bcl-xL							
	F	-6.8	-3.4	-5.9	-4.4	-11.3	-2.5
	B	-7.1	-3.0	-7.2	-3.3	-12.4	-3.5
Mcl-1							
	F	-4.7	-2.5	-4.9	-2.2	-4.0	-4.7
	B	-4.6	-3.1	-5.5	-1.5	-5.3	-2.2
Difference, Mcl-1 - Bcl-xL							
	F	2.1	0.9	1.0	2.2	7.3	-2.2
	B	2.5	-0.1	1.7	1.8	7.1	1.3

Total Ligand Grid Free Energies (LGFE, kcal/mol) for JY-1-106 binding to Bcl-xL and Mcl-1 based on the minimization, Langevin Dynamics and Explicit Solvent MD simulation approaches along with LGFE group contributions summed over the aromatic and aliphatic functional groups for Bcl-xL and Mcl-1.

Results are presented for both forward (F) and backward (B) orientations of JY-1-106 and differences (Mcl-1 – Bcl-xL) are presented for all values. Values in paranthesis for the total LGFEs are standard errors.

Additional analysis involved calculations of the LGFE scores for the aromatic and aliphatic functional groups in JY-1-106 for Bcl-xL and Mcl-1 to identify the regions of the inhibitors that 1) make the largest contribution to binding and 2) contribute to the relative binding affinities. Results in Table 1 show the LGFE for the aromatic and aliphatic groups; contributions from the hydrogen bond donors and acceptors were not significant (i.e. < 0.1 kcal/mol) and are not shown. The binding affinities are dominated by the aromatic groups in all but one case, though both the aromatic and aliphatic groups are making favorable contributions to binding. Concerning the relative binding to Bcl-xL versus Mcl-1, the aromatic groups are leading the enhanced binding to Bcl-xL in the majority of the modeling cases. These results suggest that modifications of the aromatic regions of JY-1-106 could be used to both improve affinity as well as alter the relative affinities for Bcl-xL versus Mcl-1.

### JY-1-106 disrupts complex formation between Bak and anti-apoptotic proteins in vitro and in tumor cells

**Figure 2:**
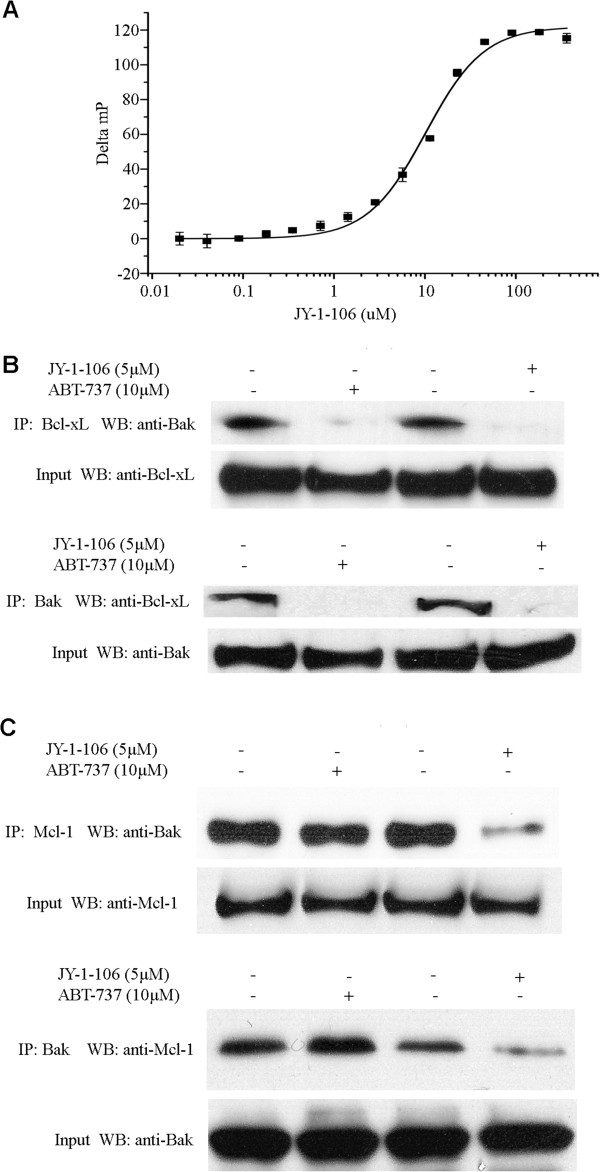
**JY-1-106 functions as Bcl-xl and Mcl-1 pan-inhibitor.** (**A**) JY-1-106 inhibits the binding of the Bak-BH3 α-helix to Mcl-1, as measured by fluorescence polarization. The sequence of the fluorescently-labeled Bak peptide is FITC-Ahx-GQVGRQLAIIGDDINR-CONH_2_. (**B**) 5 × 10^6^ REN cells were exposed to 5 μM JY-1-106, 20 μM ABT-737 or a DMSO control for 12 hours and the cells were then harvested and lysed. Bcl- x_L_ and Bak were immunoprecipitated with specific antibodies. Bak signals and Bcl- x_L_ signals were then detected by western blotting. These experiments were repeated twice. (**C**) 5 × 10^6^ REN cells were exposed to 5 μM JY-1-106, 20 μM ABT-737 or DMSO control for 12 hours and the cells were then harvested and lysed. Mcl-1 and Bak were immunoprecipitated with specific antibodies. Bak signals and Mcl-1 signals were then detected by western blotting. These experiments were repeated twice.

The modeling studies described above suggest that JY-1-106 binds to the anti-apoptotic proteins Bcl-xL and Mcl-1 in a similar fashion to that of the Bak-BH3 α-helix. We speculated that if JY-1-106 binds anti-apoptotic proteins in this way, then it should disrupt their binding to pro-apoptotic proteins. To evaluate this possibility, we first determined whether JY-1-106 disrupts the binding of Bcl-xL and Mcl-1 to Bak in vitro using fluorescence polarization (FP) assays [19]. Results show that JY-1-106 inhibits the interaction between a FITC-labeled Bak-BH3 peptide and Bcl-xL or Mcl-1 in a dose-dependent manner with IC_50_ values of 394 ± 54 nM [15] and 10.21 ± 0.83 μM (Figure 2A), respectively. The experimental *K*_i_ is about 10 times larger for Mcl-1 (*K*_i_: Bcl-xL = 179 ± 24 nM; Mcl-1 = 1.79 ± 0.15 μM, which, in terms of binding free energy, is about a 1.4 kcal/mol difference, in satisfactory agreement with the above calculations that indicate JY-1-106 binds more favorably to Bcl-xL than Mcl-1 by 2 or more kcal/mol.

IP western blotting was next applied to determine whether JY-1-106 could effectively disrupt the binding between Bak and anti-apoptotic proteins Bcl-xL and Mcl-1 in tumor cells. REN cells, a Bcl-xL and Mcl-1 overexpressing tumor cell line, were exposed to vehicle control, ABT-737 and JY-1-106, respectively. Endogenous Bcl-xL protein in these cells was immunoprecipitated and its binding to Bak was determined using anti-Bak western blotting. As demonstrated in Figure 2B, JY-1-106 as well as ABT-737 can effectively displace Bak from its inhibitory binding with Bcl-xL. The reverse IP western blotting approach, which was to immunoprecipitate Bak and immunoblot using Bcl-xL antibodies, further demonstrated that JY-1-106 can effectively disrupt the Bak–Bcl-xL protein–protein interaction. A similar blotting approach was adopted to determine the effectiveness of JY-1-106 at inhibiting the binding between Mcl-1 and Bak. As demonstrated in Figure 2C, JY-1-106, but not ABT-737, can successfully displace Bak from Mcl-1. Hence, JY-1-106 can function as a pan-Bcl-2 inhibitor.

### JY-1-106 induces cell death in cancer cells regardless of the Mcl-1 expression level

**Figure 3:**
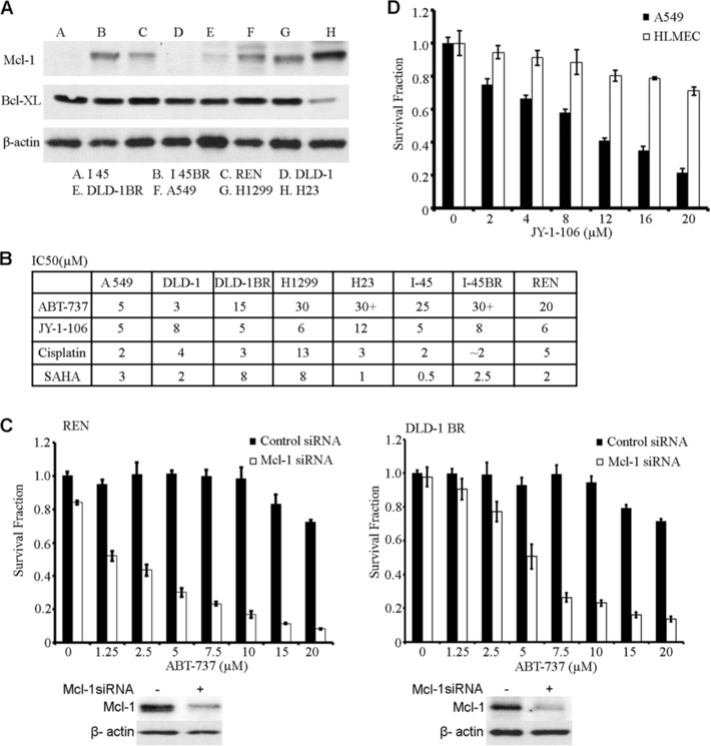
**JY-1-106 induces apoptosis in Mcl-1 overexpressed cancer cells.** (**A**) Multiple cells lines grown under regular culture conditions were collected and lysed. Bcl-xL and Mcl-1 expression in these cell lines were measured via western blotting. The β-actin expression levels were detected to normalize for protein loading. (**B**) To determine the response of A549, DLD-1, DLD-1BR, H1299, H23, I45, I45BR and REN cells to various chemotherapeutics including ABT-737, JY-1-106, cisplatin and SAHA, these cells were plated at a density of 3000 cells per well and treated with different concentrations of these drugs over a 72 hour period. An XTT assay was then performed in quadruplicate for each treatment condition. (**C**) REN and DLD-1 BR cells were transfected with control siRNA or Mcl-1 siRNA via electroporation. The cells were then seeded in 96 well plates and exposed to various doses of ABT-737. After 72 hours in culture, an XTT assay was performed to evaluate cell viability. This experiment was repeated three times. The differences between these two groups were measured using the Student’s t-test (p < 0.01). Western blotting of Mcl-1 was performed to evaluate Mcl-1 down-regulation in this experiment. (**D**) A549 and HMVEC cells were then seeded in 96 well plates and exposed to various doses of JY-1-106. After 72 hours of culture, an XTT assay was performed in quadruplicate for each treatment condition. The difference between A549 and HMVEC was compared using the Student’s t-test (p < 0.01). This experiment was repeated twice.

To determine whether JY-1-106 can induce cell growth inhibition in cancer cells with high Mcl-1 expression, the baseline protein expressions of Bcl-xL and Mcl-1 in multiple cell lines were initially examined via western blotting (Figure 3A). The results demonstrated the concurrent expression of both Mcl-1 and Bcl-xL in most of the lines, corroborating the immunostaining results in both lung and colon tumor tissues shown in Additional file 1: Figure S1. The cell lines were subsequently exposed to various chemotherapeutic agents at different doses, including cisplatin, SAHA, ABT-737 and JY-1-106. As demonstrated in Figure 3B, all the cancer cell lines that express relatively high levels of Bcl-xL and Mcl-1, and the H23 line, which shows strong Mcl-1 expression and low Bcl-xL expression, demonstrate resistance to various chemotherapy agents including cisplatin, SAHA and ABT-737. Conversely, JY-1-106 causes significant tumor cell growth inhibition in these chemotherapy-resistant cancer cell lines. Most interestingly, JY-1-106 is very effective in the I45 BR and DLD-1 BR cell lines, which are ABT-737 resistant cells established from parental I45 and DLD-1 cells.

To further assess whether JY-1-106 can overcome the Mcl-1 overexpression-related resistance to Bcl-xL inhibition, DLD-1BR and REN cells were transfected with control siRNAs or Mcl-1 siRNAs and then exposed to ABT-737. As shown in Figure 3C, after Mcl-1 reduction and ABT-737 treatment, the growth proliferation IC_50_ values for ABT-737 in these cells were improved to levels similar to those of JY-1-106 in untransfected cells (Figure 3B). Given that ABT-737 is a more potent inhibitor of Bcl-xL in vitro than JY-1-106, these data further suggest that the superior cytotoxicity of JY-1-106 is due to its pan-Bcl-2 specificity. To evaluate the potential toxicity against normal human cells, normal human microvascular endothelial cells (HMVECs) were exposed to various doses of JY-1-106. As demonstrated in Figure 3D, JY-1-106 at 5 μM has limited toxicity against HMVECs. At 20 μM, JY-1-106 caused less than 20% growth inhibition in these normal cells. TUNEL assay results demonstrated that even at 20 μM, JY-1-106 does not cause apoptosis in HMVECs (data not shown).

### JY-1-106 induces apoptosis via intrinsic apoptosis pathway

**Figure 4:**
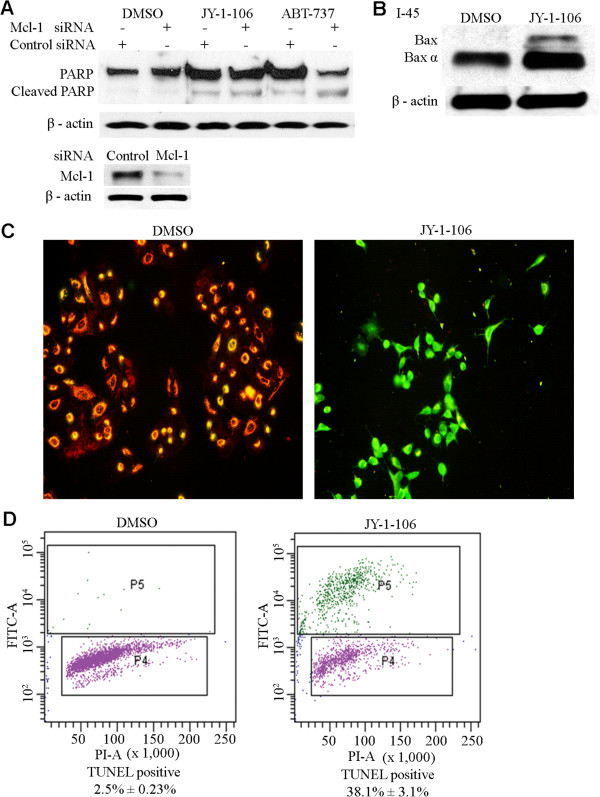
**JY-1-106 activates apoptosis via intrinsic pathway.** (**A**) 5 × 10^6^ A549 cells were transfected with Mcl-1 siRNAs or control siRNAs for 24 hours and then exposed to 5 μM JY-1-106 or 5 μM ABT-737 for 12 hours. Cleaved PARP and PARP proteins were then evaluated by western blotting using anti-PARP antibodies. Immunoblotting for β-actin was performed to normalize the loading. (**B**) 5 × 10^6^ I45 cells were exposed to 5 μM JY-1-106 or DMSO control for 12 hours. Bax and dimerized Bax were then assayed by western blotting using anti-Bax antibodies. (**C**) Determination of mitochondrial membrane potential through JC-1 staining and detection using fluorescent microscopy. I45 cells were exposed to 5 μM JY-1-106 or DMSO control for 12 hours. JC-1 dye was then loaded into the medium for the final 20 minutes of culturing. After JY-1-106 treatment, the mitochondrial membrane potential was found to be interrupted, as evidenced by the migration of JC-1 dye from the mitochondria into the cytoplasm of treated cells, and the subsequent reduction in the mitochondrial red fluorescence signals. (**D**) 2 × 10^6^ A549 cells were exposed to 5 μM JY-1-106 or DMSO control for 12 hours. These cells were then collected, fixed and subjected to a TUNEL reaction. Apoptosis signals were measured using flow-cytometry. The data shown are the average of triplicate assessments for each condition. The differences between these two groups were measured using the Student’s t-test (p < 0.01). These experiments were repeated twice.

To determine if the observed JY-1-106-induced cell growth inhibition occurred by autophagy, cultured I45 EGFP-LC-3β and A549 EGFP-LC-3β cells were established by stably transfecting EGFP-LC3β cDNA into I45 or A549 parental cells. I45 EGFP-LC-3β and A549 EGFP-LC-3β cells were treated with 5 μM JY-1-106 for 12 hours. No aggregation of EGFP-LC-3β, which indicates the formation of autophagy or LC3 cleavage, was observed by fluorescent microscopic examination or western blotting. Western blot analysis of cleaved PARP further revealed that an overnight exposure to 5 μM JY-1-106 resulted in PARP cleavage and cell death, indicating apoptosis induction. In the A549 cells, significant PARP cleavage and decreasing total PARP were observed under exposure to 5 μM JY-1-106 regardless of Mcl-1 expression. However, PARP cleavage was observed in ABT-737-treated A549 cells only upon transfection with Mcl-1 siRNA (Figure 4A). Bax–Bax dimerization after JY-1-106 treatment was observed in JY-1-106 treated I45 cells (Figure 4B).

The effects of JY-1-106 treatment on mitochondrial membrane potential were measured by JC-1 staining using fluorescence microscopy. Normally, the uptake of JC-1 dye into mitochondria results in an intense red fluorescence. When the mitochondrial membrane potential is disrupted, the JC-1 dye migrates from the mitochondria into cytoplasm and fluoresces with an intense green signal. In our current study, A549 cells were treated with JY-1-106 at concentrations of 5 μM for 12 hours. As shown in Figure 4C, a significantly reduced red fluorescence signal in mitochondria and a significantly increased green fluorescent signal in the cytosolic fraction were observed in the A549 cell line following JY-1-106 exposure.

The JY-1-106-induced apoptosis was further evaluated by a TUNEL assay. Flow cytometry was used to identify and quantify apoptotic cells in JY-1-106-treated cell suspensions. A549 cells were treated with 5 μM JY-1-106 or DMSO for 24 hours, then subjected to a TUNEL reaction and counterstained with propidium iodide. The results indicate that treatment with JY-1-106, but not with vehicle alone, results in a dramatic increase in the proportion of apoptotic cells in the treated cell suspensions (Figure 4D). Taken together, these results demonstrate that JY-1-106 induces apoptosis in tumor cells.

### JY-1-106 sensitizes tumor cells to chemotherapy and metabolic stress

**Figure 5:**
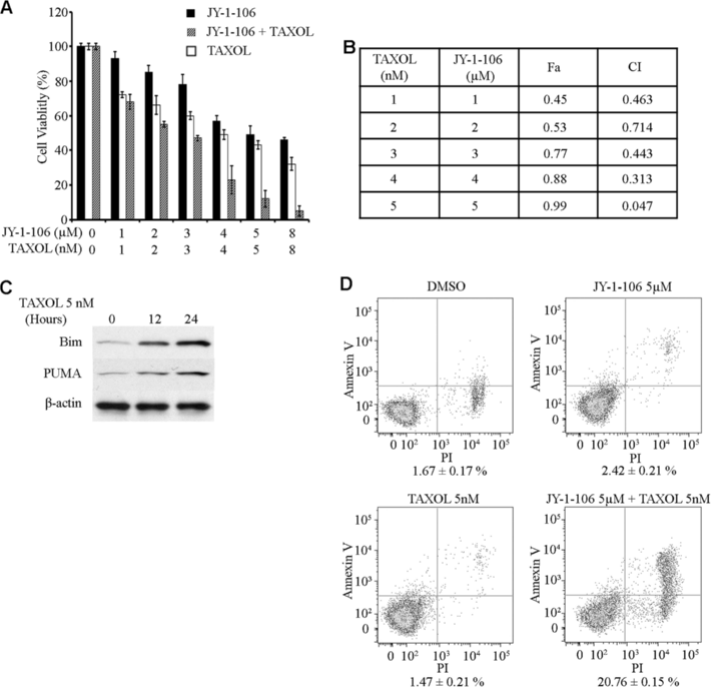
**JY-1-106 sensitized tumor cells to chemotherapy.** (**A** and **B**) Synergistic inhibition of cancer cell growth in vitro using a combination of JY-1-106 and Taxol. A549 cells were treated with JY-1-106 and Taxol at various concentrations. The viabilities of the treated cells were subsequently determined using the XTT assay. The results were converted to percentages of viable cells, and the data shown are representative of three independent experiments. Synergy was determined by calculating combination index (CI) values with Calcusyn software. (**C**) Western blot analysis of BH3-only proteins after Taxol treatment. A549 cells were exposed to 5 nM Taxol for 12 or 24 hours. Cellular proteins were then analyzed by western blotting using appropriate monoclonal and polyclonal antibodies specific for PUMA and Bim proteins. (**D**) Measurement of apoptotic cells using an Annexin V binding assay. A549 cells were labeled with Annexin V-FITC and counterstained with PI after exposure to JY-1-106, Taxol or a combination of both for 12 hours. Cells were analyzed by flow cytometry. The final results were presented as the average of three independent experiments.

To explore the therapeutic potential of JY-1-106 in conjunction with different chemotherapeutics, we evaluated the use of Taxol in combination with JY-1-106 in the A549 cell line to test for increased chemosensitivity. In the JY-1-106 treatment of A549 cells, the cytotoxic response to Taxol increased dramatically (Figure 5A). Isobologram analysis was adopted to study the potential synergism of cellular toxicity following a combination of Taxol and JY-1-106 treatment. Isobologram analysis assists in the determination of whether or not combination therapies are additive (CI = 1), synergistic (CI < 1) or antagonistic (CI > 1). The CI values presented in Figure 5B demonstrate that for all doses examined, the combinations of Taxol and JY-1-106 were synergistic in A549 cells. A similar degree of sensitization was observed in multiple cancer cell lines. Measuring BH3-only protein expression in Taxol-treated cancer cells by western blotting indicated that two BH3-only proteins, Bim and PUMA, were significantly increased upon Taxol treatments (Figure 5C), whilst others remain unchanged. Annexin-V/flow cytometric analysis of A549 cells confirmed an increased sensitization with a combination of Taxol and JY-1-106 by revealing that the percentage of apoptotic cells was significantly higher when cells were treated with both agents compared with individual treatments (Figure 5D).

**Figure 6:**
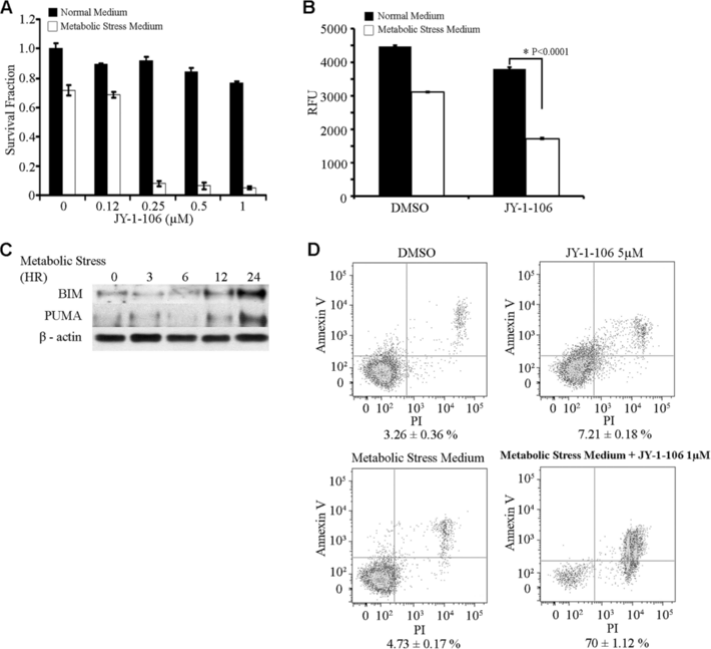
**JY-1-106 sensitizes tumor cells to metabolic stress.** (**A**) A549 cells were exposed to metabolic stress and serial doses of JY-1-106. After 72 hours, cell viability was measured using the XTT assay. The results shown are the average of triplicate assays and the experiment was repeated twice. (**B**) A549 cells were subjected to metabolic stress with or without the addition of JY-1-106 for 48 hours. The same numbers of viable cells were used for each time point. The intracellular ATP levels in these cells were measured fluorometrically. The differences between these two groups at each time point were statistically evaluated using a Student’s t-test (p < 0.01). (**C**) Western blot analysis of BH3-only proteins after metabolic stress. A549 cells were exposed to metabolic stress medium for 0, 1, 3, 6, 12 and 24 hours and analyzed by western blotting using appropriate monoclonal and polyclonal antibodies specific for PUMA and Bim proteins. (**D**) Determination of apoptotic cells with the Annexin V binding assay. A549 cells were labeled with Annexin V-FITC and counterstained with PI after exposure to JY-1-106 or metabolic stress medium or combination of JY-1-106 or metabolic stress medium for 24 hours. The cells were then analyzed by flow cytometry. The differences between combination and JY-1-106 or TAXOL were measured using the Student’s t-test (p < 0.01). The final results presented are the average of three independent experiments.

To evaluate whether inhibiting Bcl-xL and Mcl-1 could lead to decreased ATP production in metabolically stressed cancer cells, A549 cells were exposed to a very low dose of JY-1-106 in addition to metabolic stress. As demonstrated in Figure 6A, significant cell death was observed in the A549 cells treated with the combination of metabolic stress medium and 0.25 μM JY-1-106, which has little effect on cancer viability under regular culture conditions. Decreased ATP production was quantitatively measured in A549 cells (Figure 6B). Measuring BH3-only protein expression in cancer cells after metabolic stress indicated that Bim and PUMA were significantly increased upon 12 hours of metabolic stress (Figure 6C). Annexin-V/flow cytometric analysis of A549 cells again confirmed an increased sensitization with a combination of metabolic stress and 1 μM JY-1-106 by revealing that the percentage of apoptotic cells was significantly higher when cells were treated with both agents compared with individual treatments (Figure 6D).

### Inhibition of tumor growth by JY-1-106 in a lung cancer xenograft model

**Figure 7:**
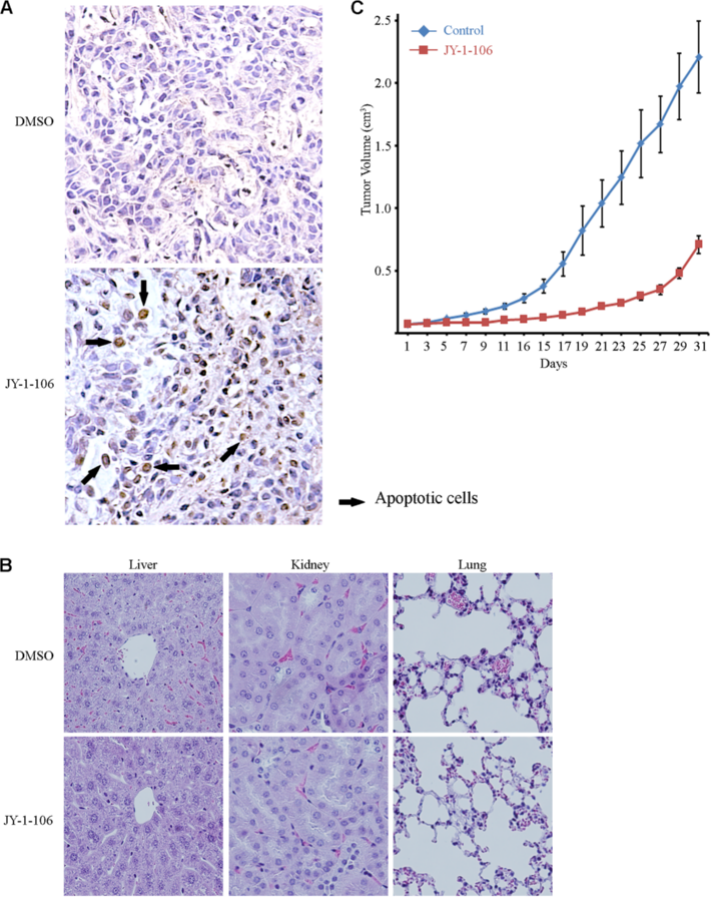
**JY-1-106 inhibits tumor growth in lung cancer xenograft model.** (**A**) Representative sections of organs and tumors collected from mice injected daily for two days with JY-1-106 i.p. at 25 mg/kg in 500 μl of PBS or with a DMSO control. Tissues were preserved in formalin and mounted in paraffin bocks. Tumor tissues were prepared using standard histology and TUNEL stained. TUNEL positive cells with brown color were indicated with arrowhead. JY-1-106 treated tumors showed elevated numbers of apoptotic cells which were detectable by an increased brown color. (**B**) Collected mouse tissues were preserved in formalin and mounted in paraffin bocks. Slides were prepared using standard histology and stained with H&E. (**C**) Suppression of tumor growth in tumor-bearing nude mice by JY-1-106. Nude mice were implanted with tumor cells using s.c. injection in the right flanks. When the tumors reached 5 mm in diameter (2 weeks after injection), JY-1-106 was administered at 25 mg/kg in 500 μl of PBS for each i.p. injection. DMSO alone was used as a mock treatment control. The injections were performed every other day for seven sequential treatments. Tumor volumes were calculated from every-other daily measurements of tumor diameters in all experimental and control groups. Results were reported as mean tumor volume ± SE over time (n = 6 mice for each treatment and control group). The difference between the tumor volumes of the JY-1-106 treated mice versus tumor volumes of the solvent control treated mice was significant (ANOVA test, p < 0.05). All of these experiments were repeated twice.

To evaluate the effects of JY-1-106 in an animal model, 10 million A549 cells were injected intraperitoneally into nude mice, and the tumors were allowed to grow for 20 days before any treatment was initiated. Following three daily intraperitoneal administrations of JY-1-106 at 25 mg/kg or vehicle control, each animal appeared to be in good health. At necropsy, no gross signs of toxicity were found. Intraperitoneally transplanted tumor samples were collected and stained using the TUNEL assay. As demonstrated in Figure 7A, JY-1-106, but not the vehicle control, induced significant apoptosis in the tumors. Histopathologic examination revealed no significant pathologic lesions in the liver, kidney, lung and spleen (Figure 7B). Chemical tests revealed normal BUN/creatinine levels in each tumor-bearing mice suggesting that no nephrotoxicity resulted from the administration of JY-1-106. Tests that evaluated liver function showed no elevation in transaminases or LDH in any of the animals. These results suggest that JY-1-106 can be administered safely as there are no significant toxicity effects.

The effects of JY-1-106 on tumor growth were further evaluated by administering this agent to nude mice bearing flank human lung cancer xenografts. Tumor-bearing mice were randomly divided into two treatment groups, a vehicle control group and JY-1-106 therapy group. The overall effects of these treatments on tumor growth were analyzed using an ANOVA statistical method. Treatment with JY-1-106 significantly inhibited tumor growth in comparison to the vehicle control (ANOVA test, p < 0.05; Figure 7C).

## Discussion

The ability of anti-apoptotic proteins to promote cancer cell survival depends on protein–protein interactions between the BH3 domains of pro-apoptotic proteins and the BH3-binding hydrophobic grooves of anti-apoptotic proteins [2,20]. This interaction is defined by the binding of the amphipathic α-helical BH3 domain from multi-BH domain proteins, such as Bax and Bak, as well as BH3 domain-only proteins, such as Bim, Bid, NOXA, Bad and PUMA, to a hydrophobic pocket formed by the BH1, BH2, and BH3 domains at the surface of anti-apoptotic proteins, such as Bcl-2, Bcl-xL and Mcl-1. In this way, the anti-apoptotic Bcl-2 proteins “neutralize” the cell-killing function of their pro-apoptotic counterparts. This interaction prompted the idea that BH3 domain mimetics may serve as potential novel anti-cancer drugs.

In this report, we characterize the novel α-helix mimetic JY-1-106 that disrupts the interactions between both Bcl-xL and Mcl-1 with Bak, which leads to apoptosis through the mitochondrial pathway in human cancer cells. Unlike several Bcl-2 antagonists such as gossypol, apogossypolone, TW-37, obatoclax, ABT-737, ABT-263, HA1–41, chelerythrine, antimycin and BHI-1, JY-1-106 was designed using an α-helix mimicry strategy involving a trisarylamide scaffold to spatially project functionality in a manner similar to that of two turns of the Bak -H3 domain α-helix. Specifically, JY-1-106 was devised to reproduce the key hydrophobic side chains of Val74, Leu78 and Ile81, all of which lie on one face of the Bak-BH3 α-helix and have been shown to be critical to mediating Bak’s protein–protein interactions [15]. Our computational modeling studies suggest that JY-1-106 binds at the hydrophobic grove of anti-apoptotic proteins such as Bcl-xL and Mcl-1 and engages amino acid residues that are involved in binding to the Bak-BH3 α-helices of pro-apoptotic proteins. The control compound JY-1-106a makes few favorable contacts leading to increased fluctuations of the binding regions of both Bcl-xL and Mcl-1, confirming that the side chains attached to the trisarylamide scaffold are required for interaction with Bcl-xL and Mcl-1. The FP assays and IP western blotting results further supported the results from our modeling study that JY-1-106 disrupts Bcl-xL–Bak and Mcl-1–Bak interactions by binding to the hydrophobic BH3-binding grooves on Bcl-xL and Mcl-1. Collectively, these data convincingly suggest that JY-1-106 is a pan-Bcl-2 inhibitor capable of antagonizing the two distinct subclasses of anti-apoptotic proteins, Bcl-2/xL and Mcl-1, both of which are critical for cancer cell survival. In fact, our animal study demonstrated that JY-1-106 is active in vivo and could selectively cause apoptosis in tumor cells and inhibit tumor growth with limited damage to normal organs.

Our present results provide new insights into the mechanisms of JY-1-106 mediated cell death. Our data suggest that JY-1-106 induces programmed cell death through the intrinsic apoptosis pathway. Pro-apoptotic Bcl-2 proteins can be classified into two main groups: multidomain pro-apoptotic proteins (Bax and Bak) and BH3-only proteins (Bid, Bim, Bad, NOXA, PUMA, BMF, BIK, and HRK) [21]. In response to death stimuli, certain BH3-only proteins, the so-called sensitizers, displace activators that include Bid and Bim from their inhibitory associations with Bcl-xL or Mcl-1. The released activators induce the activation of Bax and Bak. ABT-737 functions like the BH3 domain peptide of Bad, binding only the pro-survival Bcl-2 proteins Bcl-2 and Bcl-xL, and acts as a sensitizing, but not as an activating, BH3 stimulus [22]. As Mcl-1 can antagonize Bax activation, Mcl-1 overexpression contributes to the resistance to ABT-737 [13]. Our current results suggest that the abilities of JY-1-106 to bind both Mcl-1 and Bcl-xL contribute to Bax activation in these cancer cells. Because JY-1-106 disrupts the interaction of anti-apoptotic proteins with both of these multi-domain pro-apoptotic proteins, this compound has important advantages, since several mechanisms have been proposed for Bcl-2 family-mediated cancer cell survival including direct and indirect pathways that involve neutralization by anti-apoptotic proteins of either multi-domain or BH3-only pro-apoptotic proteins.

Our present findings clearly revealed that JY-1-106 significantly sensitizes many types of tumor cells to different chemotherapeutic agents or metabolic stress, which may, in part, be due to a restoration of apoptotic potential. Although JY-1-106 is active as a single agent in tumor cells, it may be of clinical relevance for JY-1-106 to be used in combination with commonly used chemotherapeutic drugs. It has been shown that many chemotherapeutics, including 5-FU, vinblastine, and paclitaxel, induce apoptosis by shifting the balance of proapoptotic to antiapoptotic proteins at the mitochondria [23]. Proteins containing BH3 domains are often the most dynamic participants in this process. Our current results demonstrate that both Bim and PUMA expression was induced by Taxol treatment. The resulting data indicate that the overexpression of anti-apoptotic members of the Bcl-2 family contributes to the resistance to these chemotherapeutic agents through neutralization of these BH3-only proteins, which could be overcome by using the pan Bcl-2 inhibitor JY-1-106.

We also observed that metabolically stressed cancer cells are extremely sensitive to JY-1-106 treatment, which can induce apoptosis at low dosages under these conditions. It is well-established that Bcl-2 family anti-apoptosis members protect metabolically stressed cancer cells from apoptosis by neutralizing increases in PUMA and Bim [24]. Since their BH3 domains have much higher affinities to Bcl-xL/Bcl-2 or Mcl-1, elevated PUMA and Bim levels can bind in an inhibitory manner to Bcl-xL and Mcl-1. Overexpressed Bcl-xL and Mcl-1 in cancer cells, localized at the outer membrane of mitochondria, can prevent PUMA or Bim-related Bax activation and further prevent Bax-related mitochondrial fission and apoptosis. In addition to their localization on the mitochondrial outer membrane, Bcl-xL [25] and Mcl-1 [26] were recently found to be localized within mitochondria, where they functioned to promote ATP generation rather than protect the cell against apoptosis. These new functions of Bcl-xL and Mcl-1 were further confirmed by our current observations that JY-1-106 causes significant reductions in ATP production, which would also induce cell death. These data suggest that a combination of JY-1-106 and a metabolic stress inducer could be an effective anti-cancer treatment.

## Conclusions

In summary, JY-1-106 displays single-agent activity in multiple human cancer cells and in an animal tumor model. This indicates that a strategy to disrupt protein–protein interactions via α-helix mimicry using a substituted trisarylamide scaffold was successful in developing a pan Bcl-2 family antagonist. The mechanism of cell death induced by JY-1-106 seems to be at least partially dependent upon the mitochondrial apoptosis pathway, and our current data support a process whereby this compound seems to directly activate the Bax pro-apoptotic protein. These data extend the knowledge of how BH3 agonists promote cell death in cancer cells. Towards the discovery of more potent and clinically-viable Bcl-2 antagonists, further development of BH3 mimetics, which directly activate Bax/Bak, is justified by our findings. Finally, our observations also suggest that JY-1-106 warrants further evaluation as a novel anti-cancer drug.

## Materials and methods

### Cell culture

I45 and REN (human mesothelioma cell lines), A549, H1299 and H23 (lung cancer cell lines) and DLD-1 and HCT116 (colon cancer cell lines) were purchased from the American Type Culture Collection (Manassas, VA). DLD-1, H1299, H23, I45 and REN cells were cultured in RPMI 1640 medium supplemented with 10% fetal bovine serum (FBS). A549 cells were cultured in 10% FBS-supplemented F12 medium and HCT-116 cells in 10% FBS-supplemented McCoy’s 5A medium. I45, A549, DLD-1 and H23 have doubling time of 24 hours, while REN can be doubled every 36 hours and H1299 cells can be doubled every 18 hours.

### Reagents

Cisplatin, 5-FU, Taxol and ABT-737 were obtained from Selleck Chemicals (Houston, TX). The HDAC inhibitor SAHA (suberoylanilide hydroxamic acid) was purchased from Biovision (Mountain View, CA). Rabbit antibodies against PARP, Bcl-xL and Mcl-1 were purchased from Santa Cruz Biotechnology Inc. (Santa Cruz, CA). Mouse monoclonal anti β-actin was obtained from Sigma (Saint Louis, MO).

### Molecular dynamics simulations

To study the binding of JY-1-106 to Bcl-xL and Mcl-1 at a molecular level, molecular dynamics (MD) simulations were performed using the CHARMM [27] and NAMD [28] programs with the CHARMM22 protein force field [15,25] and CHARMM General force field (CGenFF) [29,30]. Modeling and MD simulations of Bcl-xL and Mcl-1, initiated from PDB structures 1BXL and 3PK1, respectively, involved the removal of the bound peptide from each structure, the docking of JY-1-106 into the hydrophobic binding pocket on the two proteins followed by a 50 ns explicit solvent MD simulation. Both forward (NH_2_ of JY-1-106 toward the N terminus of the bound peptide) and backward orientations (vice versa) of the compound in the binding pocket were considered. A JY-1-106 analog (JY-1-106a as shown in Figure 1), which lacks the isopropoxy side chains, was also simulated with Bcl-xL and Mcl-1 to assess the importance of the hydrophobic side chains on binding.

To quantitatively interpret the binding of the two compounds, SILCS (Site Identification by Ligand Competitive Saturation) simulations [17,31] on Bcl-xL and Mcl-1 were performed. The crystal structures of the two proteins (PDBID 3PL7 and 3PK1) were solvated in a water box filled with 1 M benzene and 1 M propane followed by MD simulations. Probability distributions (FragMaps) were then used to identify regions on the protein surface that are favorable for hydrogen bond donors, hydrogen bond acceptors, aromatic groups and aliphatic groups. FragMaps were converted into GFE (grid free energy) maps. LGFE (ligand grid free energy) [32] scores were evaluated for JY-1-106 in complex with Bcl-xL and Mcl-1 using the bound ligand orientations based on three approaches that take ligand and protein flexibility into account. (1) 100 protein conformations were extracted from the SILCS simulations trajectories, and short, gas phase minimizations were performed for the docked JY-1-106 conformations with the protein fixed. The 100 minimized conformations were then used for GFE scoring. (2) 10 complex conformations were randomly selected from the first approach and a 100 ps gas phase Langevin dynamics were performed for each of the 10 conformations. During the simulation, both the ligand and all protein atoms within 8 Å of the ligand were allowed to move while other parts were fixed. 10 complex conformations were then selected from each run, resulting in 100 structures for which the GFE scores were calculated. (3) A 50 ns NPT MD simulation was conducted with explicit considerations of water for the complex and 100 structures were randomly extracted and used for the GFE scoring. Presented are total LGFE values for the full ligand and summed over all the aromatic or aliphatic side chain atoms for of the inhibitors. Errors for the total LGFE values are standard errors over the 100 conformations for each approach.

### Fluorescence polarization assay

Fluorescence polarization experiments were conducted using a BMG PHERAstar FS multimode microplate reader equipped with two PMTs for simultaneous measurements of perpendicular and parallel fluorescence emission with 485 nm excitation and 520 nm emission filters [19]. The Bak peptide was capped with fluorescein on the N-terminus and was amidated on the C-terminus (FITC-Ahx-GQVGRQLAIIGDDINR-CONH_2_). The assay was performed in a black polypropylene 384-well microplate (Costar) with a final volume of 20 μL containing varying concentrations of Mcl-1 in the presence of 15 nM FITC-Bak peptide in PBS at room temperature. The fluorescence polarization assays (FPCA) were performed using 100 nM Mcl-1 in the same buffer with varying concentrations of JY-1-106. Regression analysis was carried out using Origin (OriginLab, Northampton, MA) to fit the data to the Hill equation (1) to determine the binding affinity (*K*_d_) of Mcl-1 for the binding of the FITC-Bak peptide and to determine the IC_50_ in the FPCA. The Cheng-Prusoff equation was then used to determine the *K*_i_ for JY-1-106 as follows:

$$k_{i}=IC_{50}/\left(1+\left[L_{T}^{\mathrm{FITC}-\mathrm{Bak}}\right]/K_{d}^{\mathrm{FITC}-\mathrm{Bak}}\right)$$

IC_50_, as determined using Hill equation; [L_T_^FITC-Bak^], total ligand (15 nM FITC-Bak); *K*_d_^FITC-Bak^, 20.81 ± 0.70 nM, being the affinity of Mcl-1 for FITC-Bak peptide under the assay conditions.

### Cell proliferation assays

The effects of various inhibitors on cell viability were assessed in quadruplicate samples using the 2,3-bis(2-methoxy-4-nitro-5-sulfophenly)-5-[(phenylamino) carbonyl]-2H-tetrazolium hydroxide (XTT) assay (Trevigen, Inc. Gaithersburg, MD). Cancer cells were seeded and incubated in 96-well, flat-bottomed plates in 10% FBS-supplemented culture medium 24 hours before drug treatment. The cells were then exposed to various inhibitors at the indicated concentrations at 37°C in 5% CO_2_ for 72 hours. The medium was removed and replaced with 150 μl fresh medium containing XTT, and the cells were further cultured in the CO_2_ incubator at 37°C for 5 hours. Absorbance was determined on a plate reader at 492 nm.

### JC-1 assay

The unique cationic dye JC-1 (5, 5′6, 6′-tetrachloro-1, 1′3, 3′-tetraethylbenzimidazolylcarbocyanine iodide) was used to signal the loss of mitochondrial membrane potential [33]. Cancer cell lines were exposed to JY-1-106 at 5 μM for 12 hours. Cells were then washed with PBS and cultured with JC-1 dye for 15 minutes at 37°C in a humidified atmosphere containing 5% CO_2_. Cells were again washed with assay buffer. The loss of mitochondrial membrane potential was documented using an Olympus IX71 fluorescent microscope fitted with FITC and rhodamine filters.

### Western blotting analysis

Cancer cells were lysed using urea containing lysis buffer and equal amounts of total proteins were resolved on 4-20% Tris-glycine gels and transferred onto a nitrocellulose membrane. The membranes were then co-incubated with a rabbit anti-human Bcl-xL polyclonal antibody, a rabbit anti-human Mcl-1 monoclonal antibody, rabbit anti-human PARP polyclonal antibody, and a mouse anti-human β-actin antibody overnight. Antibody binding was then detected using chemiluminescence (Cell Signaling Technology, Danvers, MA) and signals were visualized by autoradiography.

### Apoptosis assay

After various treatments, cancer cells were detected via TUNEL assay using a FITC–TUNEL kit (Promega, Madison, WI) and then measured with BD FACSCanto II Flow cytometry. Flow cytometry data were analyzed using FlowJo software (Tree Star Corp, Ashland, OR).

### ATP assay

The Cancer cells were initially treated with metabolic stress medium with or without ABT-737 or JY-1-106 for up to 24 hours. ATP was measured using the Fluorometric ATP Assay Kit (Biovision, San Francisco, CA).

### Evaluation of JY-1-106 *in vivo*

Approval to conduct this study was obtained from the Institutional Animal Care and Use Committees (IACUC) at the Scott and White Memorial Hospital Texas Health Science Center. This study was conducted in compliance with institutional IACUC and NIH guidelines. To evaluate the efficacy of JY-1-106, 2 × 10^6^ A549 cells were injected into the flank of female nude mice (6 weeks old). Once the transplanted tumor reached 5 mm in diameter, mice were treated with vehicle solution or JY-1-106 (25 mg/kg, i.p., every other day for 2 weeks for a total of 7 injections). Tumor sizes were measured three times per week until reaching 1.5 cm in diameter. To further assess the immediate effect of JY-1-106 *in vivo*, mice that had flank tumors were injected i.p. with JY-1-106 (25 mg/kg) or vehicle solution. Twenty-four hours after injection, the spleen, liver, heart, lung and flank tumors were collected, fixed and hematoxylin and eosin stained. Apoptosis in these samples was determined using the TUNEL assay.

### Statistical analysis

Continuous variables were compared using the Student’s t test. The therapeutic relationship between JY-1-106 and Taxol was assessed with the CalcuSyn program, based on the principle of Chou and Talalay. In the Chou and Talalay method, the concentration-effect curve is linearized by logarithmic transformation as follows:

$$\log\left(\mathrm{fu}–1–1\right)=\log\left(\mathrm{fa}–1–1\right)–1=\mathrm{nlog}\left(C\right)–\mathrm{nlog}\left(\mathrm{Cm}\right),$$

fu is the fraction of cells left unaffected after drug exposure; fa is the fraction of cells affected by the exposure; C is the drug concentration used; Cm is the concentration that achieves the median effect; and n is the curve shape parameter. Cm and n are equivalent to the IC_50_. The values of n (obtained from the slope), nlog(Cm) (obtained from the absolute value of the intercept), and, therefore, Cm are obtained by plotting log(fu-1 - 1) versus log(C).

The program returns the CI (combination index) values that are indicative of synergism, additive effects, or antagonism between two agents. CI analysis provides qualitative information on the nature of drug interactions, and CI, a calculated numerical value, also provides a quantitative measure of the extent of drug interaction. A CI of less than, equal to, and more than 1 indicates synergy, additivity, and antagonism, respectively.

### Immunohistochemistry

Formalin-fixed, paraffin-embedded tissues of lung adenocarcinoma and colon adenocarcinoma were examined for the expression of Mcl-1 and Beclin-1 proteins. All samples were histologically confirmed and de-identified. Approval to conduct this study was obtained from the Institutional Ethics Review Board at the Scott and White Memorial Hospital Texas Health Science Center. This study was conducted in compliance with the Helsinki Declaration. The human colon cancer samples were stained using an avidin-streptavidin-biotin-peroxidase kit (Vector Laboratories).

### Consent

Written informed consent was obtained from the patient for publication of this report and any accompanying images.

## Abbreviations

ANOVA: Analysis of variance; BH3: Bcl-2 homology domain 3; BH3: Bcl-2 homology domain 3; LGFE: Ligand Grid Free Energies; SILCS: Site identification by ligand competitive saturation; siRNA: Small interfering RNA; TUNEL: Terminal deoxynucleotidyl transferase dUTP nick end labeling; SD: Standard deviation.

## Competing interests

ADM Jr. has a financial interest in the company SilcsBio LLC, which is marketing the SILCS technology. The remaining authors declare that they have no competing interests.

## Authors’ contributions

XC, RS and SF conceived and designed study; analyzed and interpreted data; drafted and revised the manuscript. JY and KJ synthesized the JY-1-106 compound. DJ performed the statistical analyses. WY and KV and AM performed molecular dynamics simulations. SF and PTW conducted the FP assay. CP and AR and JH carried out XTT assay and apoptosis assays. RT and MR performed Flow-cytometric tests. HP helped animal experiments and lab tests and examined the animal samples. All authors read and approved the final manuscript.

## Supplementary Material

Additional file 1: Figure S1 Mcl-1 and Bcl-xL expression patterns in lung and colon adenocarcinomas. Lung adenocarcinoma and colon adenocarcinoma tissue array slides were stained for Bcl-xL and Mcl-1 proteins using the ABC staining kit from Vector Lab. The co-existence of Mcl-1 and Bcl-xL expression in tumor cells on the tissue slides was assessed using the Fisher exact and Chi-square tests. Within each tumor sample, both Mcl-1 and Bcl-xL expression in adjacent normal tissues was very low. Neither Mcl-1 nor Bcl-xL expression was detected in the control normal tissues included in these tissue arrays. Click here for file
