# Supplementary figures and images for: The novel BH3 α-helix mimetic JY-1-106 induces apoptosis in a subset of cancer cells (lung cancer, colon cancer and mesothelioma) by disrupting Bcl-xL and Mcl-1 protein–protein interactions with Bak
Source: Mol Cancer. 2013 May 16;12:42. doi: 10.1186/1476-4598-12-42 (PMC3663763; doi:10.1186/1476-4598-12-42)

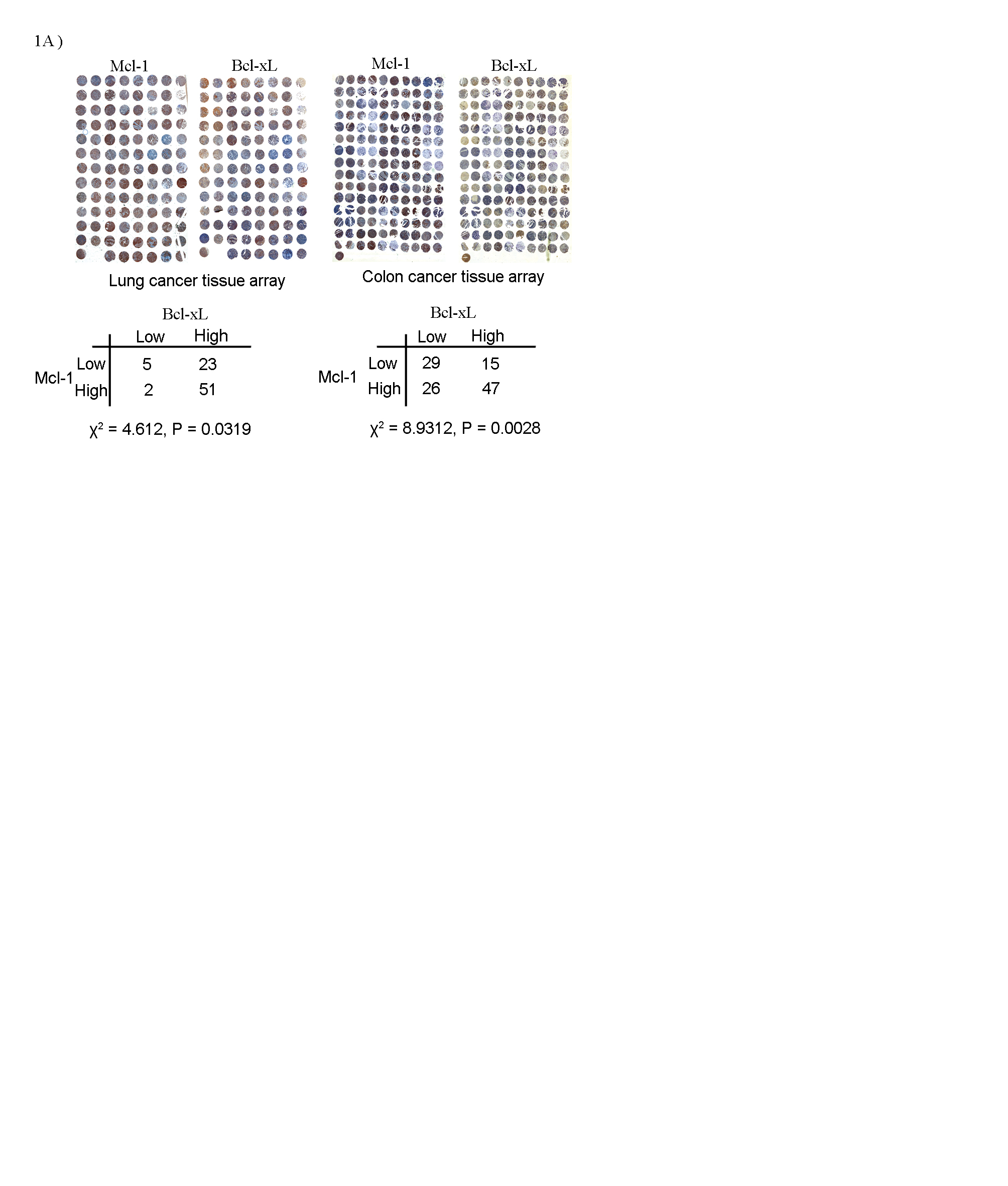

Supplement: Additional file 1: Figure S1 — Mcl-1 and Bcl-xL expression patterns in lung and colon adenocarcinomas. Lung adenocarcinoma and colon adenocarcinoma tissue array slides were stained for Bcl-xL and Mcl-1 proteins using the ABC staining kit from Vector Lab. The co-existence of Mcl-1 and Bcl-xL expression in tumor cells on the tissue slides was assessed using the Fisher exact and Chi-square tests. Within each tumor sample, both Mcl-1 and Bcl-xL expression in adjacent normal tissues was very low. Neither Mcl-1 nor Bcl-xL expression was detected in the control normal tissues included in these tissue arrays. [file 1476-4598-12-42-S1.jpeg]
